# Shifts in biomass and structure of habitat‐formers across a latitudinal gradient

**DOI:** 10.1002/ece3.7714

**Published:** 2021-05-27

**Authors:** Talia Peta Stelling‐Wood, Alistair G. B. Poore, Paul E. Gribben

**Affiliations:** ^1^ Evolution & Ecology Research Centre UNSW Sydney Sydney NSW Australia; ^2^ Centre of Marine Science and Innovation UNSW Sydney Sydney NSW Australia; ^3^ Sydney Institute of Marine Science Mosman NSW Australia

**Keywords:** climate change, habitat structure, latitudinal gradient, macroalgae, morphological variation, trait

## Abstract

Global patterns of plant biomass drive the distribution of much of the marine and terrestrial life on Earth. This is because their biomass and physical structure have important consequences for the communities they support by providing food and habitat. In terrestrial ecosystems, temperature is one of the major determinants of plant biomass and can influence plant and leaf morphology. In temperate marine systems, macroalgae are major habitat‐formers and commonly display highly variable morphology in response to local environmental conditions. Variation in their morphology, and thus habitat structure on temperate reefs, however, is poorly understood across large scales. In this study, we used a trait‐based approach to quantify morphological variability in subtidal rocky reefs dominated by the algal genus *Sargassum* along a latitudinal gradient, in southeastern Australia (~900 km). We tested whether large‐scale variation in sea surface temperature (SST), site exposure, and nutrient availability can predict algal biomass and individual morphology. We found *Sargassum* biomass declined with increasing maximum SST. We also found that individual morphology varied with abiotic ocean variables. Frond size and intraindividual variability in frond size decreased with increasing with distance from the equator, as SST decreased and nitrate concentration increased. The shape of fronds displayed no clear relationship with any of the abiotic variables measured. These results suggest climate change will cause significant changes to the structure of *Sargassum* habitats along the southeastern coast of Australia, resulting in an overall reduction in biomass and increase in the prevalence of thalli with large, highly variable fronds. Using a space‐for‐time approach means shifts in morphological trait values can be used as early warning signs of impending species declines and regime shifts. Consequently, by studying traits and how they change across large scales we can potentially predict and anticipate the impacts of environmental change on these communities.

## Graphical abstract

Using a trait‐based approach, we tested predictions about how the biomass and morphological traits of macroalgae from the genus *Sargassum* varied across six degrees of latitude on the southeastern coast of Australia. Our results suggest climate change will cause significantly changes to *Sargassum* habitat along the southeastern coast of Australia, resulting in an overall reduction in biomass and increasing the prevalence of thalli with large, highly variable fronds.

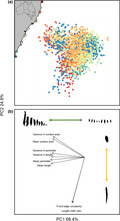

## 1 INTRODUCTION

Global patterns of plant biomass and primary production have shaped the distribution of much of the diversity and abundance of life on Earth (Huston & Wolverton, 2009). Both aquatic and terrestrial plants are important as primary producers (trophic role) and as habitat‐formers (structural role). They form complex, usually three‐dimensional structures that facilitate other organisms through the provision of resources such as food, living space, and refuge (Bertness et al., 2001; Wright & Gribben, 2017). Thus, they have strong positive effects on associated communities by enhancing abundance and species richness (Bruno et al., 2005; Romero et al., 2015). Given that many habitat‐forming species have broad distributions that cover large environmental gradients, understanding how their structural attributes change across this scale is a critical first step in determining how habitat‐formers control community structure across large spatial scales.

Gradients in abiotic variables play an important role in shaping broad‐scale patterns in habitat‐formers (e.g., Benedetti‐Cecchi, 2001; Crain et al., 2004; Marzinelli et al., 2015; Parrish & Bazzaz, 1982). Terrestrial plant biomass, for example, is highly correlated with latitude, meaning that plants are generally taller in the tropics than in mid‐ and low‐latitude regions (Moles et al., 2009). These patterns are linked to higher mean annual temperatures and increased precipitation in tropical climate regions. Similar patterns have been found in subtidal marine habitats, where size (Mabin et al., 2013) and canopy cover of kelps (Marzinelli et al., 2015) vary predictably with temperature. In contrast, Lloyd et al. (2020) documented strongly nonlinear relationships between biomass and temperature for intertidal algae measured over ~1,300 km. However, despite the nonlinear relationship, sea surface temperature was still found to be a major contributor to these patterns, in combination with anthropogenic disturbance (Lloyd et al., 2020). Temperature is, however, not the only factor known to influence algal biomass, with other factors such as wave action and nutrient concentration also known to correlate with biomass (Kraufvelin et al., 2010).

Large‐scale variability in environmental conditions can also influence the morphology of individual habitat‐formers. For example, leaf size in terrestrial plants is well known to vary with latitude, with leaf size decreasing with distance from the equator (Royer et al., 2005). Similarly, leaves from plants found in tropical regions typically have smoother edges, compared with those in temperate areas, which are generally more dissected, toothed, or serrated (Traiser et al., 2005). These patterns are commonly correlated with temperature and precipitation (Dong et al., 2020); however, other variables such as light availability and nutrients can also influence leaf morphology (Jurik et al., 1982). Comparatively, few studies have investigated large‐scale patterns in the morphology of macrophytes. A recent study by Voerman et al. (2019) did, however, show that frond length and width in the green alga *Caulerpa filiformis* decreased with increasing latitude. Similarly, Clark et al. (2018) found *Hormosira banksii* individuals from warmer, edge populations were smaller in size and had smaller vesicles than those from cooler, central populations. Instead, much of the research on morphological variability in macroalgae has been limited to small‐scale studies or manipulative experiments. For example, the red alga *Chondrus crispus* develops more complex fronds when grown at 20°C compared with 5°C (Kübler & Dudgeon, 1996). Similarly, experimental manipulation of light intensity led to morphological variability in the red alga *Asparagopsis armata* (Monro & Poore, 2005), while different flow rates and water depths can also cause macroalgae to develop distinct morphotypes (Haring & Carpenter, 2007).

The high morphological variability displayed by macroalgae means intraspecific variability is often high, both among individuals within populations and even among parts within individuals (intraindividual) (Stelling‐Wood et al., 2020). Many terrestrial plants can produce leaves of different sizes and shapes within a single plant, in response to the environmental conditions that part of the plant experiences (e.g., heterophylly; Valladares et al., 2000). Similarly, in aquatic plants aerial leaves and submerged leaves are not only often totally different shapes, but also differ physiologically, with submerged leaves lacking both stomata and a cuticle (Wells & Pigliucci, 2000). This suggests that small‐scale variation in the abiotic conditions experienced by plants can have potentially large effects on their morphology. Thus, predicting the effects of environmental change on macroalgae will depend on an understanding of the relative roles of factors operating across multiple spatial scales, that is, from local to regional.

In this study, we used a large‐scale survey to determine patterns of biomass and morphological variation in the dominant macroalgae from the genus *Sargassum* across six degrees of latitude on the southeastern coast of Australia. Australia's temperate reef, the “Great Southern Reef” (Bennett et al., 2016), covers more than 71,000 km^2^ of the temperate Australian coastline and is a global biodiversity hot spot. Much of this coastline supports taxonomically diverse underwater forests dominated by macroalgae, with most of the literature to date focused on “true kelps” from the order Laminariales (Coleman & Wernberg, 2017). Less well understood is the ecology of the abundant, habitat‐forming species in the brown algal order Fucales (Coleman & Wernberg, 2017). Of the fucoids, *Sargassum* is the most species‐rich genus, comprising both temperate and tropical species. The densely branched *Sargassum* provide three‐dimensional habitat for diverse assemblages of invertebrates and fish, as well as epiphytic and understory algae (Chen et al., 2020). Species from the genus *Sargassum*, however, are characterized by extremely high morphological variability, which makes species identification difficult (Coleman & Wernberg, 2017), and much of the ecological research to date has focused on *Sargassum* at the genus level. Previous research has found *Sargassum* cover correlates with temperature (Wernberg et al., 2011), but the importance of local environmental conditions in determining *Sargassum* biomass and morphology has not been tested (Coleman & Wernberg, 2017). Quantifying how fucoids on subtidal rocky reefs vary across important environmental gradients will be critical to understanding how these habitat‐formers and their associated communities will respond to environmental change (Coleman & Wernberg, 2017).

The ocean climate off southeastern Australia is driven predominantly by a strong western boundary current, the East Australia Current (EAC). The EAC brings warm, nutrient‐poor water from the Coral Sea down to the Tasman Sea and Southern Ocean (Godfrey et al., 1980), resulting in a strong latitudinal gradient in ocean temperature and nutrient concentrations. Given this, our aims were to quantify (a) biomass and (b) morphological variability within the genus *Sargassum*, using traits that describe frond morphology, in order to (c) identify which abiotic variables (temperature, nutrients, and exposure) were most important for predicting large‐scale patterns in the morphological variability of *Sargassum* along a latitudinal gradient. Using latitude as a proxy for temperature, we hypothesize that *Sargassum* biomass would positively correlate with latitude and that frond size would negatively correlate with latitude.

## 2 METHODS

### 2.1 Study area and species

**FIGURE 1:**
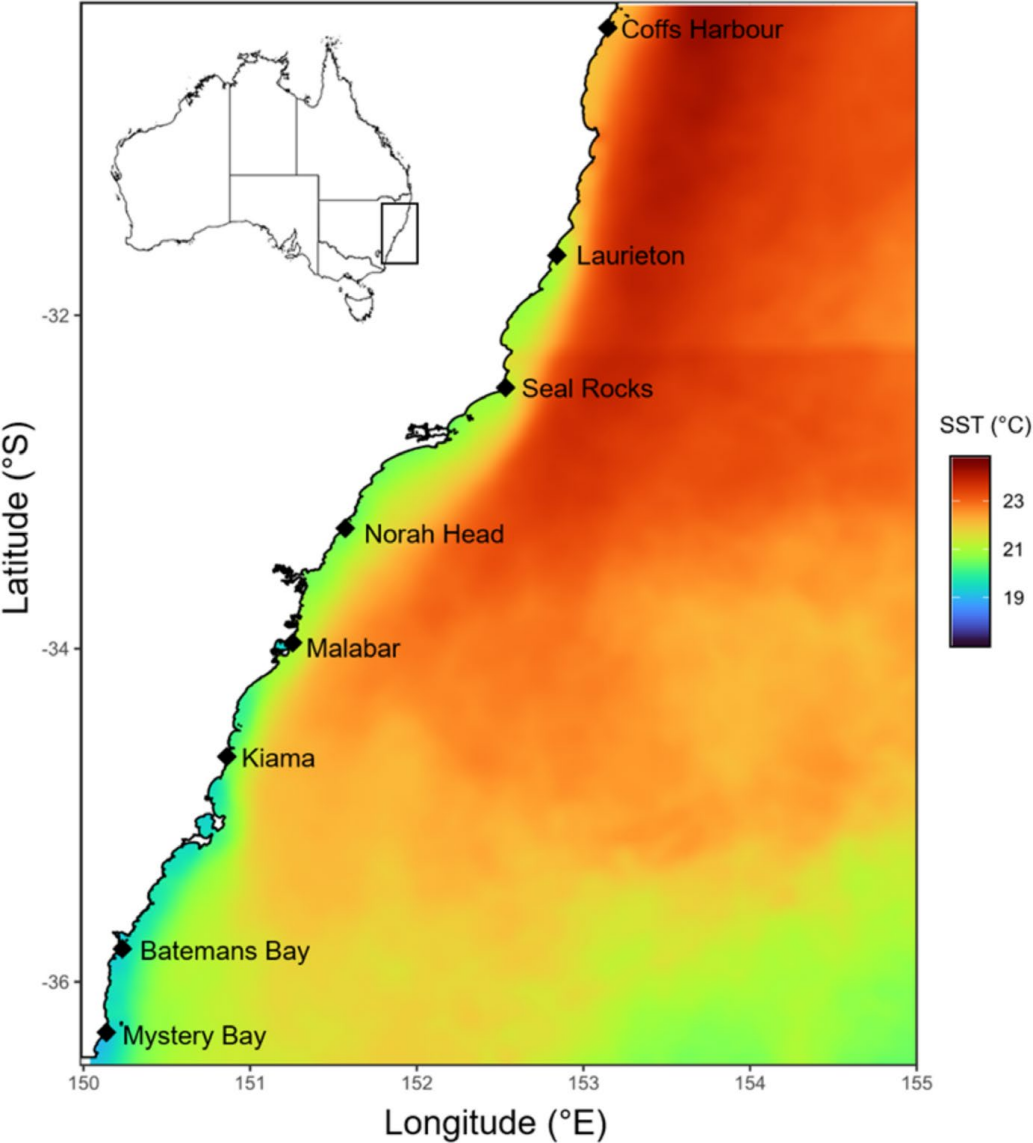
Location of all eight sites on the southeastern coast of Australia. Ocean color reflects annual mean temperature. All temperature data were SST obtained by remote sensing

During October 2017, we surveyed *Sargassum* biomass and morphology at eight sites along ~900 km of the southeastern coast of Australia: Coffs Harbour (−30.28°S, 153.15°E), Laurieton (−31.65°S, 152.84°E), Seal Rocks (−32.44°S, 152.53°E), Norah Head (−33.28°S, 151.57°E), Malabar (−33.97°S, 151.26°E), Kiama (−34.67°S, 150.86°E), Batemans Bay (−35.8, 150.23°E), and Mystery Bay (−36.6°S, 150.14°E) (Figure 1). Directly offshore, the ocean climate along the east coast of Australia is dominated by the EAC, which is typically ~30 km wide and 200 m deep and flows up to 4 knots (2 m/s^2^) (Suthers et al., 2011). This results in a gradient in sea surface temperature (SST) of 2–4°C on average, from north to south along this coastline (i.e., SST decreases with latitude) (Table S.1). This range, however, increases significantly when considering extremes in SST (maximum and minimum), with temperatures as high as 28°C in the north and as low at 10°C at the southernmost site (Table S.1).

Species of *Sargassum* generally form mixed subsurface canopies with thalli growing 10–2 m in height (Womersley, 1987). *Sargassum* are generally foliose and bushy, with reproductive upper parts of the thallus morphologically distinct from the lower perennial portions of the plant, which have wider basal fronds. There is only limited knowledge of the distribution of individual species of *Sargassum* along the Australian coastline. There are approximately 19 species of temperate *Sargassum*, many of which are thought to span the entire coastline of Australia (Coleman & Wernberg, 2017). Along the NSW coast, at the time of writing, the Atlas of Living Australia (http://www.ala.org.au) has 471 occurrence records for the genus *Sargassum*, 355 of which are not identified beyond genus. Given most species within the genus are morphologically similar and that thalli are highly variable within and among individuals (Stelling‐Wood et al., 2020), we did not distinguish among species within the genus *Sargassum*. Instead, we quantified the structure of *Sargassum* habitats by collecting all individuals belonging to the genus *Sargassum* that fell within our quadrats.

### 2.2 Survey design

At each site, six 5‐m transects were randomly placed perpendicular to shore in areas where *Sargassum* habitat was present. Transects were placed in the shallow subtidal at depths between 1 and 3 m at low tide and were spaced at least 10 m apart (in any direction) to ensure independent sampling. Along each transect, five marks were randomly positioned with flagging tape and all *Sargassum* within a 25 × 25 cm quadrat at each mark was removed, placed into bags and frozen for later processing in the laboratory.

### 2.3 Quantifying algal biomass and morphology

Frozen algae were thawed, and the total wet biomass of *Sargassum* was recorded from each quadrat. One intact thallus (i.e., holdfast present and all branches still attached) was randomly selected from each quadrat for a measure of maximum height and biomass of an individual. Remaining algal material was then cut into branches, with branches acting as the replicate unit for all other morphological traits measured. Twenty branches were randomly selected from each quadrat and eight frond morphology traits quantified from each branch (Stelling‐Wood et al., 2020). These were frond size traits (mean frond surface area, mean frond length, mean frond width), frond shape traits (mean frond shape complexity as estimated by mean perimeter/√surface area and mean length:width ratio), and traits that measured intraindividual variance in morphology (variance in frond surface area, variance in frond length, and variance in frond width). Frond traits were measured using image analysis software ImageJ (1.50i; National Institute of Health, Bethesda, MD, USA) from images of 10–15 fronds randomly removed from each branch and scanned using a Canon LIDE220 A4 Flatbed Scanner.

### 2.4 Environmental conditions at study sites

We estimated SST, nutrient concentration, and exposure at each study site. Daily maps of satellite‐derived SST data were used to generate temperature profiles for each of the study sites. These were obtained from the “Advanced Very High‐Resolution Radiometer” (AVHRR) aboard the “National Oceanic and Atmospheric Administration” (NOOA) series of satellites, archived by the “Integrated Marine Observing System” (IMOS) and the Australia Bureau of Meteorology (BOM). We used IMOS–SRS Satellite–SST L3S‐01 day composite–day and nighttime composite, as day + nighttime SST composites are considered a good compromise between data availability and resolution. All datasets were accessed from the Australian Ocean Data Network (AODN). At each site, a bounding box 20° × 20° in size (~20 × 20 km) was selected with the left side of the box bounding the site and then measuring 20° east from there to minimize the box including nonoceanic pixels. From this, long‐term temperature profile datasets were extracted covering one entire year (October 2016–October 2017). All temperature measurements from each pixel within the bounding box over the full time period were included, and from this mean, SST was calculated for each site. Maximum SST and minimum SST were taken as the highest and lowest SST recorded for any pixel within the bounding box at each site. Maximum SST and minimum SST only were used in further analyses as these represent the most stressful conditions for algae and therefore the most likely to cause morphological variability.

Mean concentrations of dissolved macronutrients (nitrate and phosphate) were extracted for each site from mean annual data from CSIRO Atlas of Regional Seas data (CARS, version 2009). These data are available at 0.5° resolution, and as such, a bounding box of 0.5° × 0.5° size was used around each site. Data from 0 to 5 m deep were averaged for each site.

Aspect was used as an approximation of exposure at each site, as the prevailing large swell and storm direction are from the south–southeast along the eastern coast of Australia (Kulmar, 1995; Short & Trenaman, 1992). Wave conditions were assumed to be consistent across our study regions based on consistent extreme wave heights (varying from eight to nine meters across NSW) and direction (southeast to south–southeast in central to southern NSW) (Shand et al., 2011). Aspect was quantified as a continuous variable from 0 to 1, indicating the direction faced by the shoreline nearest to the site, where 1 represents a shoreline facing directly south–southeast, that is, maximum exposure, and 0 represents a shoreline facing the opposite direction (north–northwest), that is, protected from prevailing large swell (Turnbull et al., 2018).

### 2.5 Statistical analyses

Thallus size measurements (biomass and maximum height) can be confounded by differences in the age of algal individuals and genetic and/or environmentally driven differences in growth rates and are therefore not considered functional traits. For this reason, they were excluded from all trait analyses in this study and were analyzed separately. Therefore, using only frond morphology traits, variation in *Sargassum* morphology among and within sites was visualized using principal component analysis (PCA) with the “princomp” function in R. The PCA was based on a correlation matrix with all trait values log‐transformed, scaled, and centered around zero to ensure each variable had equal contribution to the PCA.

For the morphological traits, we generated a covariation matrix to test for colinearity between individual traits. The matrix identified several covarying traits (Figure 3b). As a result, we used the PCA to convert correlated variables into a set of unrelated composite values (the principal components; Song et al., 2013). Using this method, we found the first two axes of the PCA (i.e., the first two principal components) explained over 93% of the variation, and therefore, the scores from these two axes were used as response variables in all further analyses. PC1 represented a univariate proxy for frond size and intraindividual variance in frond size traits (i.e., the variance in frond size traits that occurs within a single thallus) (Figure 3b), and PC2 represented a univariate proxy for frond shape (Figure 3b). Scores from both axes were tested against latitude and abiotic variables: maximum SST, minimum SST, aspect (proxy for exposure), nitrate concentration, and phosphate concentration. In these models, “site” and “sample ID” (a unique identifier for each quadrat) were included as random factors, as multiple branches were measured from each quadrat. For significant relationships, we used parametric bootstrapping with 500 simulations to establish 95% confidence intervals around each predicted model line.

We then used backward stepwise model selection to determine which abiotic variable(s) were the best predictors of *Sargassum* morphology. To do this, we used the scores from the first two principal components as before against response variables (latitude and abiotic variables). Each model included “site” and “sample ID” again as random factors. A covariation matrix showed nitrate and phosphorous concentrations had a pairwise correlation greater than 0.9, and as such, only nitrate concentration was used in the model. The initial (full) model was simplified by backward selection, sequentially removing nonsignificant variables until all remaining variables were significant. The best model was then selected based on AIC value (lowest AIC).

To determine the robustness of these results, we also analyzed the data with random forest algorithms (Breiman, 2001). Random forests produce a measure of predictor variable importance by determining the deterioration of the predictive ability of the model when each predictor is replaced in turn by random noise. The resulting deterioration is a measure of the importance of each predictor to model accuracy. Tree‐based algorithms such as random forests have different assumptions to linear modeling, and consistency between predictors of various methods can be used to gauge confidence in results.

In separate analyses, each of individual thallus biomass and thallus maximum height was tested against latitude and these same abiotic environmental variables using LMMs with “site” were included as a random factor. LMMs were also used to test total biomass of *Sargassum* per quadrat against latitude and those same abiotic variables, with “site” as a random factor. For these models, biomass was log‐transformed and only quadrats that contained algae were included (i.e., empty quadrats were excluded).

All linear mixed models were conducted using the lme4 package (Bates et al., 2015). Where appropriate, models were validated by inspecting residual plots for heteroskedasticity (Zuur et al., 2009). The random forest analysis was done using the “random forest” package in R (Liaw & Wiener, 2002). All statistical analyses were undertaken in R v3.4.2 (R Core Team, 2018).

## 3 RESULTS

### 3.1 Abiotic environmental variables

Overall, maximum and minimum SST decreased with increasing latitude; however, the relationship between maximum SST and latitude was only borderline significant (Figure S.1a & S.1b). Nitrate and phosphate concentrations displayed the opposite pattern, with concentrations significantly increasing with latitude (Figure S.1c & S.1d). Site exposure was highly variable among sites and followed no clear pattern with latitude (Figure S.1e).

### 3.2 Large‐scale variation in *Sargassum* biomass

**FIGURE 2:**
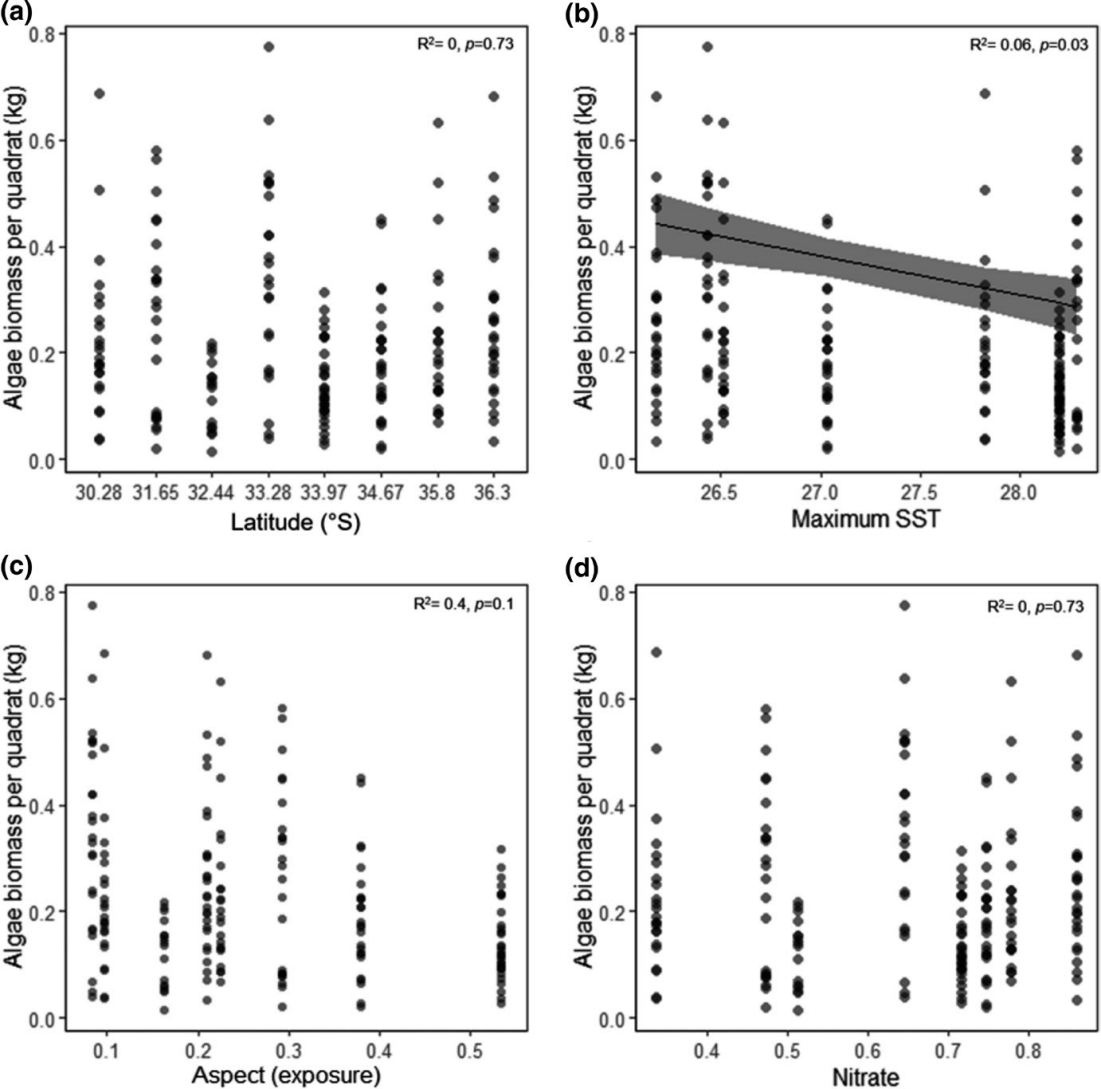
Relationship between total Sargassum biomass per quadrat (kg per 0.0625 m^2^) and (a) latitude, (b) maximum SST (°C), (c) aspect (proxy for site exposure), and (d) mean annual nitrate concentration (mmol N/m^3^). Significant relationships were modeled using linear regression, shown as the black line, with 95% confidence intervals in gray

*Sargassum* biomass was highest at Norah Head (−33.28°S) at 4.25 kg/m^2^ and was lowest at the site just north of this, Seal Rocks (−32.44°S) at 1.04 kg/m^2^ (Figure S.2). The total biomass of *Sargassum* per quadrat was highly variable within and among sites and did not correlate with latitude, aspect (proxy for site exposure), or macronutrients (Figures 2a,c,d, Table S.2). Total *Sargassum* biomass per quadrat was negatively correlated with maximum SST (Figure 2b, Table S.2); however, maximum SST explained little of the variation in algal biomass (*r* ^2^ = 0.06; Table S.2).

### 3.3 Large‐scale variation in *Sargassum* morphology

In line with total biomass per quadrat, thallus size (thallus biomass and maximum height) did not correlate with latitude (Table S.3). Thallus height was, however, negatively correlated with maximum SST (Figure S.3 & Table S.3) and positively correlated with phosphate concentration (Figure S.4, Table S.3). Thallus biomass did not significantly correlate with any abiotic variables (Table S.3).

**FIGURE 3:**
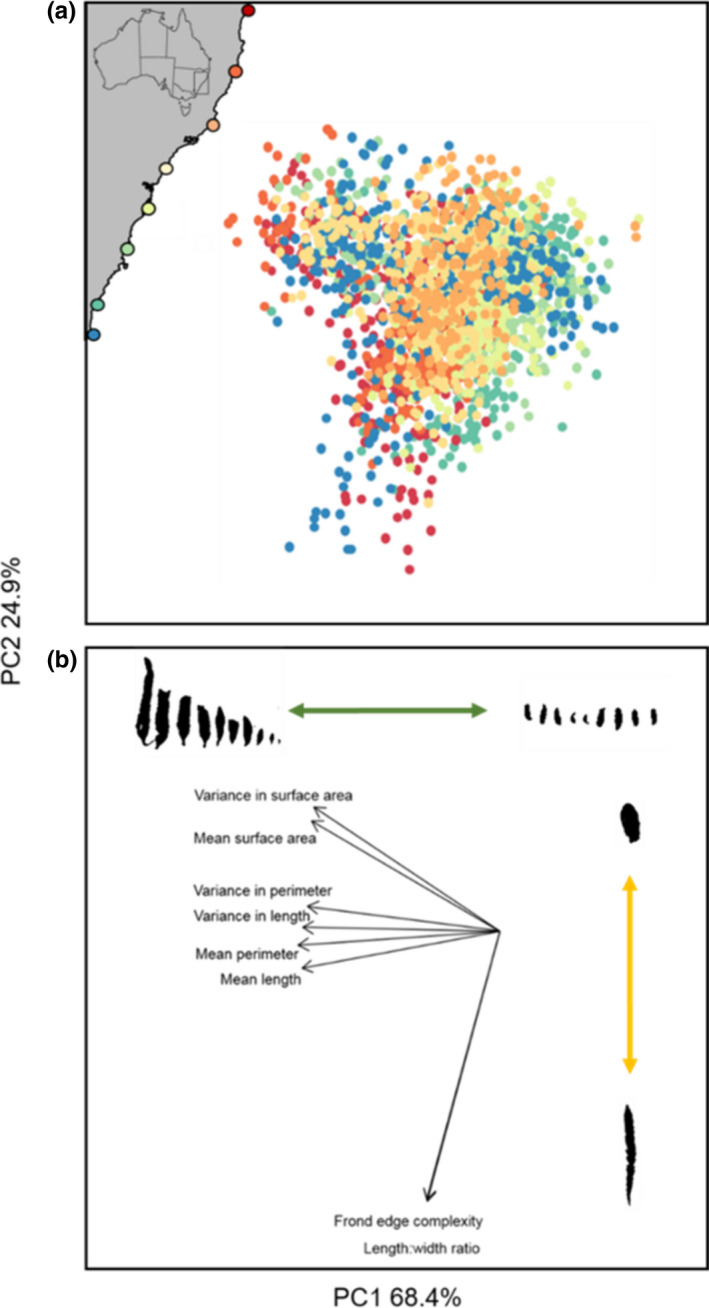
(a) PCA visualizing the morphological variation of *Sargassum* spp. for all sites, including map inset with geographical location of sites color‐coded from the lowest latitude site (30.28°S) in red through to the highest latitude site (36.3°S) in blue. (b) Biplot showing relationship between frond traits and the first two principal components. Colored arrows correspond to morphological trait variation captured by each principal component, with the green arrow representing frond size and intraindividual variance in frond size traits (PC1) and yellow arrow representing frond shape traits (PC2)

The morphology of *Sargassum* was highly variable both within and among sites. In the PCA, the first two components explained 93.3% of the morphological variation: 68.4% by PC1 and 24.9% by PC2 (Figure 3). PC1 was most correlated with frond size and the variance in those frond size traits (hereafter intraindividual variation in frond size) (Figure 3b): mean frond length (−0.407), mean frond perimeter (−0.415), variance in frond length (−0.406), and variance in frond perimeter (−0.396). PC2 was highly correlated with length:width ratio (−0.646) and frond shape complexity (−0.642), representing frond shape traits (Figure 3b).

**FIGURE 4:**
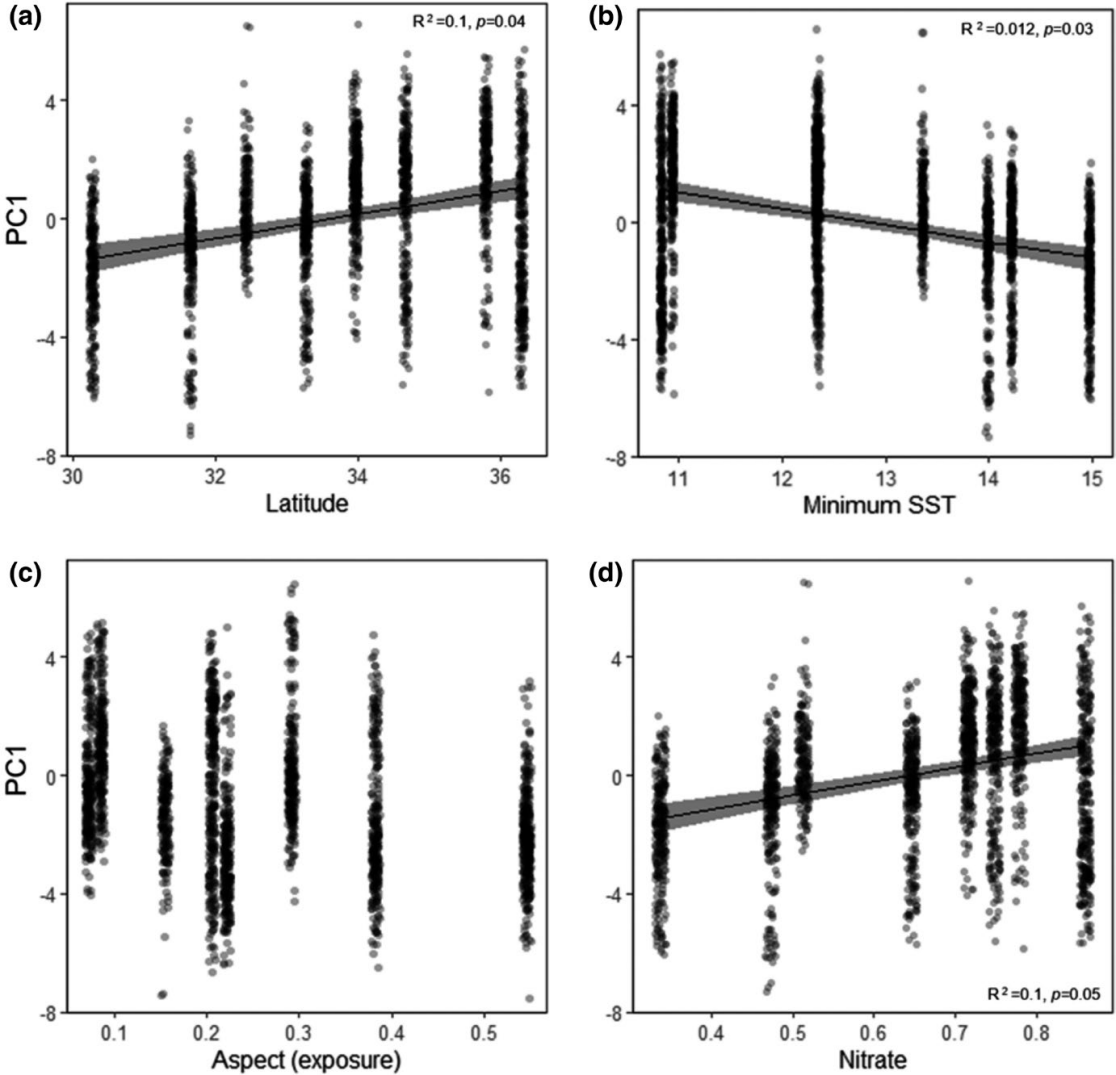
Relationship between frond size and intraindividual variance in frond size traits (PC1) and a selection of abiotic variables. (a) Latitude (°S), (b) minimum SST (°C), (c) aspect (proxy for site exposure), and (d) mean annual nitrate concentration (mmol N/m^3^). Significant relationships were modeled using LMMs with “sample ID” and “site” as random factors, shown as the black line, with 95% confidence intervals in gray

PC1 was positively correlated with latitude (Figure 4a, Table S.3), indicating that frond size and intraindividual variance in frond size decreased as latitude increased. PC1 showed no relationship with maximum SST (Table S.3) but was negatively correlated with minimum SST (Figure 4b, Table S.3), with frond size and intraindividual variance in frond size decreasing with increasing latitude. PC1 did not correlate with aspect (proxy for site exposure) (Figure 4c, Table S.3). Similarly, PC1 showed a positive relationship with nitrate concentration (Figure 4d, Table S.3) and was not correlated with phosphate concentration (Table S.3). All abiotic variables again, however, explained little of the variance in PC1 (all *R* ^2^ ≤ 0.1, Table S.3). PC2 was not significantly correlated with any of the abiotic variables measured (Table S.3).

Model selection identified PC1 (frond size and intraindividual variance in frond size) was best predicted by nitrate concentration (Table 1, M1: *F*_1,5_ = 3.91, *p* = 0.05). However, the model containing maximum SST and nitrate and the full model containing all abiotic variables were within 2 AIC values of the best model, suggesting they were similar in performance to the best model. When these two models were tested, however, they were not found to be significantly different from the best model (Table 1, M2: *F*_1,6_ = 3.05, *p* = 0.08). Similarly, no significant differences were found between any of the other models and the best model (Table 1, M3: *F*_3,8_ = 3.5, *p* = 0.32, M4: *F*_2,7_ = 3.4, *p* = 0.18), indicating maximum SST, minimum SST, and aspect were not significant in predicting Sargassum morphology. Random forest analysis confirmed this (variance explained 24.44%), with nitrate ranking as the most important variable and maximum SST ranking as the next most important variable (Table S.4).

**TABLE 1:** Model selection table using LMMs to predict frond size and intraindividual variance in frond size traits (PC1), with “sample ID” and “site” as random effects

Model	Max SST	Min SST	Aspect	Nitrate	AIC	Δ AIC
**M1**				**x**	**5,150.5**	**0**
M2	x			x	5,151.3	0.8
M3	x	x	x	x	5,151.8	1.3
NULL					5,153.8	3.3
M4	x	x		x	5,153.9	3.4

Note

Best model is shown in bold (M1). ΔAIC indicates the difference in model parsimony as explained by AIC relative to the best model; lower ΔAIC values indicate higher support for the model.

PC2 (frond shape) was best predicted by maximum SST, aspect, and nutrients (Table S.5, M1: *F*_3,7_ = 9.6, *p* = 0.02). However, again, the full model also including minimum SST was within 2 AIC values, suggesting similar performance. When these two models were tested, they were found to be significantly different (Table S.5, M2: *F*_1,8_ = 4.7, *p* = 0.03), suggesting minimum SST also plays a role in predicting frond shape. The random forest model identified nitrate concentration as the most important variable when predicting PC2 (Table S.6); however, the random forest model only explained 5.33% of the variance in frond shape, and as such, these results should be viewed with caution.

## 4 DISCUSSION

Understanding how habitat‐forming organisms vary across large spatial scales is important for predicting how habitats will respond to environmental change. Across a gradient of six degrees of latitude (~900 km), from subtropical to cool temperate sites, we found total biomass per quadrat and the morphology of the habitat‐forming alga *Sargassum* were correlated with ocean temperature and nutrient availability, despite very high levels of variation within sites and even within individual thalli. Although we did not find support for our first hypothesis that *Sargassum* biomass would increase with increasing latitude, it was correlated with maximum SST. We did find support for our second hypothesis, with frond size negatively correlating with latitude. However, frond size traits were highly correlated with variance in frond size traits meaning that intraindividual variability covaried with frond size. Consequently, while algal individuals from low‐latitude sites tended to have on average larger fronds, these fronds also tended to be more variable in size. In comparison, at high‐latitude sites, the reverse was true with algal individuals having on average smaller fronds that were more uniform in size. These findings suggest both small‐ and large‐scale environmental factors are important in driving morphological variability in *Sargassum*, and highlight that any changes to the ocean climate along the southeastern coast of Australia could have dramatic effects on the structure of these habitats.

### 4.1 Large‐scale variation in *Sargassum* biomass

Algal biomass per quadrat was highly variable both among sites and within sites. Interestingly, across our sites we found no evidence for latitudinal patterns in *Sargassum* biomass. Lloyd et al. (2020) also found weak relationships between biomass and latitude for five out of six intertidal algae. Similarly, latitudinal patterns in biomass were absent for *C. filiformis* (Voerman et al., 2019). The EAC is the dominant current within our study region; however, the current itself is highly variable. Around ~33°S, it separates from the coastline, periodically producing eddies that separate from the EAC and meander southward. These eddies cause sporadic upwelling and downwelling events along the coast and can have strong impacts on the ocean climate within the region. This was evident in our ocean climate data and suggests that within our study region, latitude cannot be used as a proxy for abiotic variables. This nonlinear gradient in ocean climate is possibly the reason we did not detect any latitudinal patterns in the biomass of *Sargassum*. Latitudinal patterns have, however, been found in other marine macrophytes, with kelp cover known to positively correlate with latitude (Marzinelli et al., 2015). These inconsistencies highlight the need for more large‐scale surveys in order to better understand patterns biomass production in macrophytes.

*Sargassum* biomass per quadrat was, however, found to significantly decline with increasing maximum SST. Interestingly, thallus biomass did not correlate with SST or any other environmental variable. This suggests that the biomass of individual thalli was not driving the large‐scale patterns in total *Sargassum* biomass documented here, and instead, variation in the number and/or density of thalli may be responsible for these patterns. Temperature is a strong predictor of terrestrial plant biomass, with biomass negatively correlating with temperature (Lin et al., 2010). In contrast, marine primary producer biomass usually follows the opposite pattern, with biomass increasing as temperature decreases (Huston & Wolverton, 2009; Marzinelli et al., 2015). The significant negative relationship between *Sargassum* biomass and SST found in this study contrasts with a study undertaken on the western coast of Australia where *Sargassum* biomass was instead found to increase with increasing SST (Wernberg et al., 2011). The maximum temperatures recorded in the west coast study were, however, considerably lower (~25°C) than those recorded on the east coast in our study (~28°C). Interestingly, the site in our study with the highest standing stock of algal biomass was Norah Head (−33.28°S), which had an usually low maximum SST of 26.4°C, much lower than surrounding sites to the north and south (~2°C lower). As a result, this site had a temperature regime much more similar to those found by Wernberg et al. (2011) in Western Australia. Temperatures of 26–27°C and above have been found to induce mortality in *Sargassum linearfolium* (one of Australia's most widespread temperate fucoid species; Coleman & Wernberg, 2017) (Bui et al., 2018), suggesting that the exceptionally warm maximum temperatures on the east coast may be close to *Sargassum's* thermal limit.

### 4.2 Large‐scale variation in *Sargassum* morphology

We documented extensive variability in the morphology of *Sargassum*, among sites, within sites, and within individual branches of algae. The morphological traits of *Sargassum* fronds tended to form two distinct clusters when plotted in “trait space.” PC1 was highly correlated with those traits that quantified frond size and intraindividual variance in frond size, while PC2 was correlated with traits that quantified frond shape. Frond size and intraindividual variance in frond size were the biggest source of morphological variability, alone capturing 68.4% of the variation. Thalli tended to have larger, more variable fronds at low‐latitude sites, shifting to small, less variable fronds as latitude increased. It was particularly interesting to see such a large extent of the morphological variability described here occurring within individuals, as even among terrestrial plant studies, the variability that occurs among the structural features within an individual habitat‐former is rarely considered (but see Bruschi et al., 2003). Furthermore, while intraindividual variability has been documented in several species of brown algae before (Stelling‐Wood et al., 2020), this is the first time it has been found to correlate with a large‐scale environmental gradient.

The latitudinal patterns in algal frond size found here match those of terrestrial plant systems, whereby leaf size is on average larger in low‐latitude regions and decreases with distance from the equator (Royer et al., 2005; Wright et al., 2017). On land, this pattern is largely driven by latitudinal variability in rainfall and temperature (Wright et al., 2017). While water availability is not limiting for subtidal macroalgae, frond size and intraindividual variance in frond size were highly correlated with maximum SST supporting experimental research, which has found a relationship between frond morphology and temperature (e.g., Kübler & Dudgeon, 1996). Across larger scales, however, the relationship between frond morphology and temperature appears to be less predictable. In *C. filiformis*, frond length is negatively correlated with latitude (Voerman et al., 2019), while *H. banksii* appear to display an opposing trend, with individuals from warmer, edge populations having larger vesicles than those individuals from cooler, central populations (Clark et al., 2018). These inconsistencies suggest that macroalgae may differ from terrestrial plants with species‐specific responses overriding general broad‐scale patterns. Alternatively, for many of these studies temperature was inferred from latitude, and as such, local‐scale variability in ocean temperatures or other factors that may interact with temperature cannot be ruled out.

Frond size and intraindividual variance in frond size were also negatively correlated with nitrate concentration, with thalli generally having small, uniformly sized fronds at sites with high nitrate concentrations, shifting to large, more variable fronds as nutrient levels decreased. This relationship was, however, only borderline significant, despite the fact that model selection identified the best model for predicting frond size and intraindividual variance in frond size as containing only nitrate concentration. Previous research has shown filamentous and branched macroalgal forms have higher nutrient uptake than more simple forms, due to their higher surface area‐to‐volume ratio (Wallentinus, 1984). Surface area‐to‐volume ratios will, however, generally be highest for small fronds meaning that this ratio was highest for algae at sites with high nutrient concentrations. Given this, its unlikely frond size‐related nutrient uptake is driving the patterns in frond size found in this study.

In addition to frond size, frond shape also varied substantially among and within our study sites. Variance in frond shape accounted for ~25% of the morphological variation we documented, with fronds varying from elongated and highly serrated to short, wide, and a smooth edge (Figure 3b). In terrestrial plants, leaf shape is known to correlate with climate. Trees in cool, wet climates often produce leaves that are more lobed or have a more serrated edge (Peppe et al., 2011). We did not find these same patterns in macroalgal frond shape. Instead, frond shape did not correlate with any of the abiotic variables measured. Thermoregulation is usually recognized as the major driver of variation in terrestrial plant leaf shape (Nicotra et al., 2011); therefore, it is surprising that these same patterns did not exist in macroalgae.

None of the traits used to quantify *Sargassum* morphology correlated with site exposure (measured as aspect). This was somewhat unexpected as wave exposure is well documented to influence algal morphology in other species of macroalgae (Hurd, 2000). For example, Johnson and Koehl (1994) found *Nereocystis luetkeana* grown at sites with strong currents had long, flat and narrow blades, whereas those grown at protected sites had significantly wider, more ruffled blades. Similarly, vesicle size in *H. banksii* was larger in estuarine environments compared with those from the open ocean (Ralph et al., 1998). The effects of wave action on *Sargassum* have not been previously investigated. However, the most exposed site in our study was only 0.54, which was halfway between fully protected (0) and fully exposed (1), so perhaps the wave action at our sites was not strong enough to drive shifts in the morphology of *Sargassum*. Alternatively, these inconsistencies may be in response to the use of different methods to assess wave exposure in these studies.

While relationships clearly exist between the structure of *Sargassum* and large‐scale abiotic conditions, heterogeneity within sites suggests local‐scale conditions (on scales of 10s of meters) also play a role. Reef topography can influence algal assemblages, with more complex topographies thought to increase microhabitat availability and lead to higher variability within algal assemblages (Toohey et al., 2007). Similarly, small‐scale heterogeneity in nutrient supply could also drive within‐site variability if individual macroalgae experience long periods of nutrient limitation (Smith, 1986). Miller et al. (2020) found local‐scale variability in the thermal tolerance of *H. banksii* embryos was related to small‐scale genetic differences. Alternatively, variation among different species of *Sargassum* could explain within‐site variability. Hwang et al. (2004) found varying seawater temperatures were closely related to *Sargassum* occurrence; however, they found high variability between different species. They suggested temperature limitations and nutrient utilization strategies could vary between even closely related species (i.e., within the same genus), thereby driving this variability. Due to the difficulties in identifying *Sargassum* to species (Coleman & Wernberg, 2017), we did not classify algae to species; therefore, it is possible this within‐site variability is due to species‐specific responses to temperature.

### 4.3 Habitat provisioning in a changing ocean

The ecological function of habitat‐formers is closely tied to their morphology (Bishop et al., 2013; Peeters, 2002; Stelling‐Wood et al., 2020). We found the morphology of the dominant habitat‐forming macroalgae from the genus *Sargassum* varied predictably with latitude. Current climate models predict a further strengthening of the EAC off the eastern coast of Australia, which will bring warmer, nutrient‐poor water further down the coast. Our results suggest this will cause significant changes to *Sargassum* habitat along this stretch of coastline, resulting in an overall reduction in total biomass and increasing the prevalence of thalli with large, highly variable fronds. Previous research has found a negative relationship between these morphological traits and the abundance of associated epifauna (Stelling‐Wood et al., 2020), meaning future conditions will likely result in fewer invertebrates colonizing these algal habitats. These epifaunal communities represent an important trophic link between primary producers and higher tropic levels, and consequently, any change to these communities could have significant cascading effects to higher trophic levels (Chen et al., 2020).

Using a space‐for‐time approach means shifts in morphological trait values can be used as early warning signs of impending species declines or even regime shifts on temperate reefs (Baruah et al., 2019). Thus, by examining traits and how they change rather than simply the presence or absence of species we will not only able to better quantify ecosystem functioning, but we can also potentially predict and anticipate the impacts of environmental change on these communities before species losses occur (Mouillot et al., 2013). The study of traits and how they vary also potentially offers a way to unify terrestrial and marine ecosystems; if similar traits correlate with similar functions in the two systems, trait‐based approaches could assist in the development of generalizable principles that can cross ecosystem boundaries.

## CONFLICT OF INTEREST

No competing interests.

## AUTHOR CONTRIBUTIONS

**Talia Peta Stelling‐Wood:** Conceptualization (equal); Formal analysis (lead); Investigation (lead); Methodology (equal); Writing‐original draft (lead); Writing‐review & editing (equal). **Alistair G. B. Poore:** Conceptualization (equal); Formal analysis (supporting); Methodology (supporting); Supervision (equal); Writing‐review & editing (equal). **Paul E. Gribben:** Conceptualization (equal); Formal analysis (supporting); Methodology (supporting); Supervision (equal); Writing‐review & editing (equal).

## Supporting information

Supplementary Material

## Data Availability

Data are archived on the Research Data Australia (RDA) https://doi.org/10.26190/mppw‐3a36.
