# Supplementary material for: Shifts in biomass and structure of habitat‐formers across a latitudinal gradient
Source: Ecol Evol. 2021 May 27;11(13):8831–42. doi: 10.1002/ece3.7714 (PMC8258212; doi:10.1002/ece3.7714)
Supplement: Supplementary file 1 — Supplementary Material [file ECE3-11-8831-s001.docx]

Supplementary material

Table S.1. Ocean temperature characteristics of each site. All temperature data were sea surface temperature obtained by remote sensing.

| Temperature characteristic | Coffs Harbour | Laurieton | Seal Rocks | Norah Head | Malabar | Kiama | Batemans Bay | Mystery Bay |
| --- | --- | --- | --- | --- | --- | --- | --- | --- |
|  | -30.28°S | -31.65°S | -32.44°S | -33.28°S | -33.97°S | -34.67°S | -35.8°S | -36.3°S |
| *1 year*  *(2016 – 2017)* |  | | | | | | | |
| Mean (°C ± SE) | 21.64 ± 0.015 | 20.92 ± 0.014 | 21.08 ± 0.012 | 20.25 ± 0.013 | 20.60 ± 0.013 | 20.01 ± 0.014 | 18.82 ± 0.016 | 18.78 ± 0.017 |
| Minimum (°C) | 14.97 | 13.99 | 13.35 | 14.21 | 12.33 | 12.35 | 10.94 | 10.82 |
| Maximum (°C) | 27.81 | 28.28 | 28.19 | 26.43 | 28.19 | 27.03 | 26.51 | 26.18 |
| n | 19349 | 21947 | 25336 | 19911 | 21783 | 20349 | 21048 | 21619 |


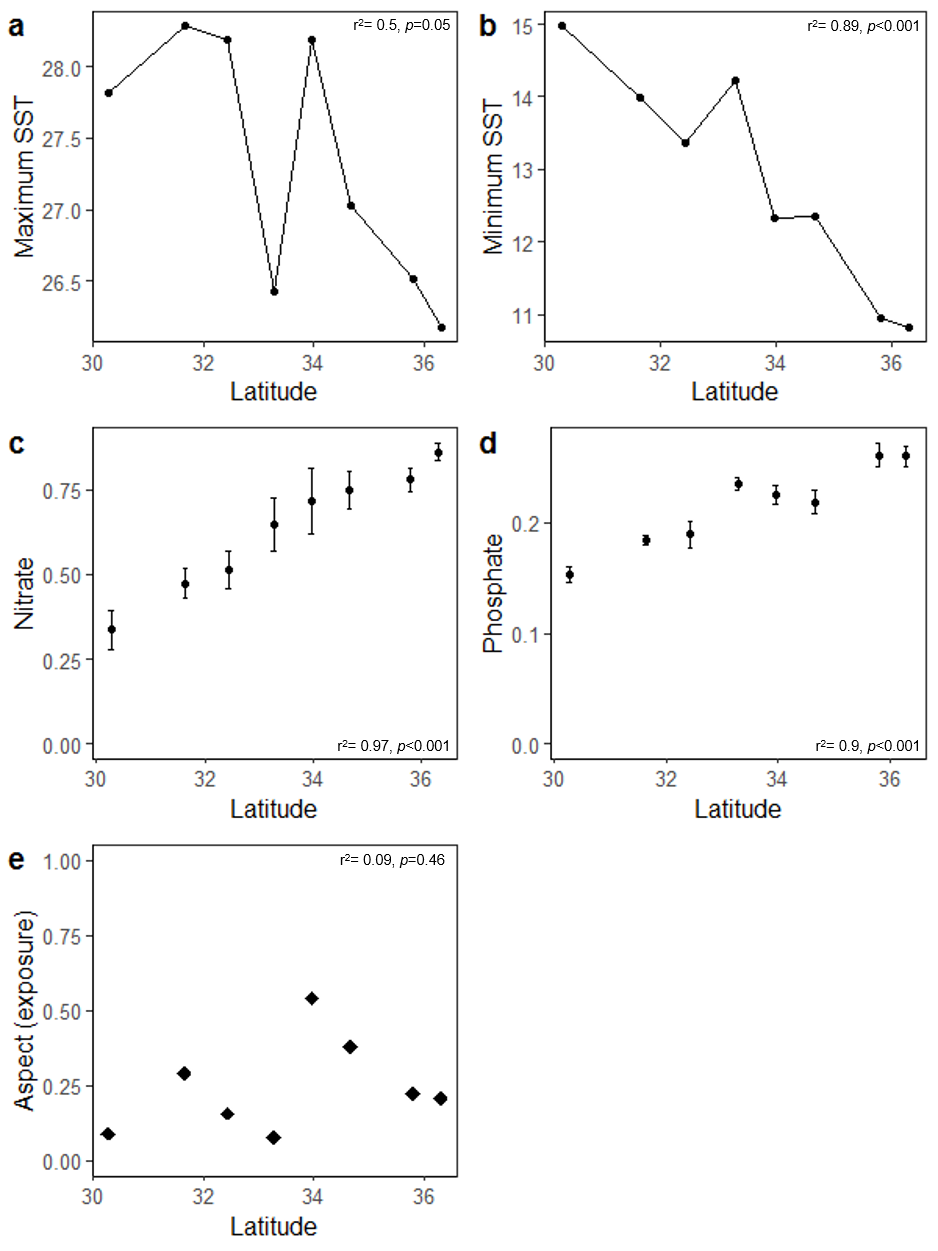


Figure S.1. Abiotic variables against latitude. (a) Maximum SST (°C), (b) minimum SST (°C), (c) mean nitrate concentration (mmol N m^-3^), (d) mean phosphate concentration (mmol N m^-3^), and (e) aspect (proxy for site exposure). Nutrient measurements are annual averages averaged for 0-5m deep.


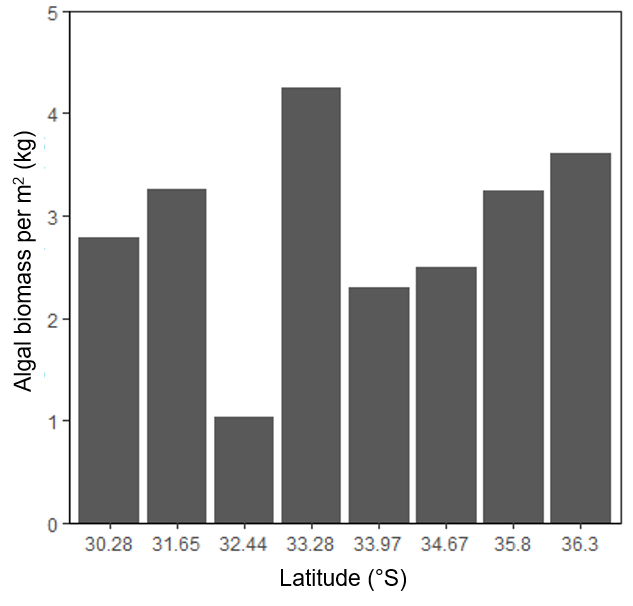


Figure S.2. Total *Sargassum* biomass collected from each site standardised by total area sampled (kg/m^2^). Sites are identified here by their latitude.


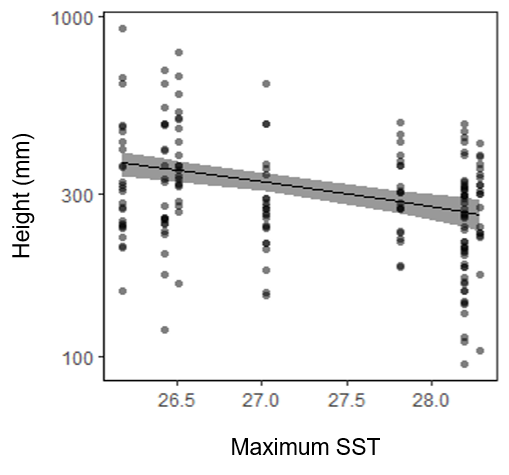


Figure S.3. Relationship between thallus height and maximum SST (°C). Y-axis has been log transformed. Significant relationships were modelled using LMM with ‘site’ as a random factor, shown as the black line, with 95% confidence intervals in grey.


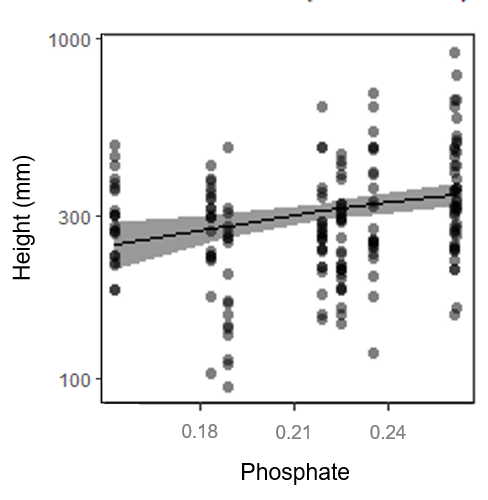


Figure S.4. Relationship between thallus height and mean annual phosphate concentration (mmol N m-3). Y-axis has been log transformed. Significant relationships were modelled using LMM with ‘site’ as a random factor, shown as the black line, with 95% confidence intervals in grey.

Table S.2. LMMs for algal biomass per quadrat against latitude and abiotic variables. Biomass units is kg per quadrat (0.0625m^2^). Biomass was log transformed and ‘site’ was included as a random factor. Significant predictors are indicated with *.

|  | Minimum SST | | | Maximum SST | | | Mean nitrate | | | Mean phosphate | | | Site exposure | | | Latitude | | |
| --- | --- | --- | --- | --- | --- | --- | --- | --- | --- | --- | --- | --- | --- | --- | --- | --- | --- | --- |
|  | R^2^ | F | P | R^2^ | F | P | R^2^ | F | P | R^2^ | F | P | R^2^ | F | P | R^2^ | F | P |
| Biomass | 0 | 0.04 | 0.83 | 0.06 | 4.87 | 0.03* | 0 | 0.12 | 0.73 | 0.01 | 0.78 | 0.38 | 0.4 | 2.62 | 0.1 | 0 | 0.12 | 0.73 |

Table S.3. LMMs for algal traits against latitude and abiotic variables. For principle components ‘site’ and ‘sample ID’ were included as random factors. For thallus size traits (biomass and height) only ‘site’ was included as a random factor. Significant predictors are indicated with *.

|  | Minimum SST | | | Maximum SST | | | Mean nitrate | | | Mean phosphate | | | Site exposure | | | Latitude | | |
| --- | --- | --- | --- | --- | --- | --- | --- | --- | --- | --- | --- | --- | --- | --- | --- | --- | --- | --- |
|  | R^2^ | z | P(z) | R^2^ | z | P(z) | R^2^ | z | P(z) | R^2^ | z | P(z) | R^2^ | z | P(z) | R^2^ | z | P(z) |
| PC1 | 0.1 | 4.7 | 0.03* | 0 | 0.1 | 0.8 | 0.1 | 3.9 | 0.05* | 0.09 | 3.1 | 0.07 | 0.1 | 2.9 | 0.09 | 0.1 | 4.1 | 0.04* |
| PC2 | 0 | 1.1 | 0.3 | 0 | 1.2 | 0.3 | 0.01 | 2.3 | 0.1 | 0.01 | 2.6 | 0.1 | 0 | 0.13 | 0.7 | 0.01 | 2.3 | 0.13 |
| Biomass | 0 | 0.05 | 0.82 | 0 | 3.7 | 0.06 | 0 | 0.01 | 0.9 | 0 | 0.62 | 0.4 | 0.05 | 3.3 | 0.07 | 0 | 0.05 | 0.8 |
| Height | 0 | 1.83 | 0.18 | 0.1 | 8.5 | 0.003* | 0.04 | 2.03 | 0.15 | 0.06 | 4.2 | 0.04* | 0.01 | 0.61 | 0.4 | 0.05 | 2.6 | 0.1 |

Table S.4. Variable importance rankings from random forest regression model predicting frond size and variability in frond size (PC1). ‘*MSE*’ is mean square error and represents a decrease in the predictive ability of the model (i.e. an increase in *MSE*) when that given variable is replaced by random noise.

| Abiotic variable | % increase MSE |
| --- | --- |
| Maximum SST | 33.6 |
| Minimum SST | 33.4 |
| Aspect (proxy for exposure) | 30 |
| Mean nitrate concentration | 34.2 |

Table S.5.  Model selection table using LMMs to predict frond shape (PC2), with ‘sample ID’ and ‘site’ as random effects. Best model is shown in bold (M1). ΔAIC indicates the difference in model parsimony as explained by AIC relative to the best model; lower ΔAIC values indicate higher support for the model.

| Model | Max SST | Min SST | Aspect | Nutrients | AIC | **Δ** AIC |
| --- | --- | --- | --- | --- | --- | --- |
| **M1** | **x** |  | **x** | **x** | 7230.4 | **0** |
| M2 | x | x | x | x | 7231.7 | 1.3 |
| NULL |  |  |  |  | 7233.3 | 2.9 |

Table S.6. Variable importance rankings from random forest regression model predicting frond shape (PC2). ‘*MSE*’ is mean square error and represents a decrease in the predictive ability of the model (i.e. an increase in *MSE*) when that given variable is replaced by random noise. The model, however, only explained 5.33% of the variance in frond shape and as such these results should be viewed with caution.

| Abiotic variable | % increase MSE |
| --- | --- |
| Maximum SST | 26.9 |
| Minimum SST | 30.2 |
| Aspect (proxy for exposure) | 27.2 |
| Mean nitrate concentration | 32.6 |
